# An algorithm to detect unexpected increases in frequency of reports of adverse events in EudraVigilance

**DOI:** 10.1002/pds.4344

**Published:** 2017-11-16

**Authors:** Luis C. Pinheiro, Gianmario Candore, Cosimo Zaccaria, Jim Slattery, Peter Arlett

**Affiliations:** ^1^ European Medicines Agency London UK

**Keywords:** abuse, adverse drug reaction, EudraVigilance, European Medicines Agency, medication error, misuse, pharmacoepidemiology, pharmacovigilance databases, quality defect, signal detection, time‐series forecasting

## Abstract

**Purpose:**

The European Medicines Agency developed an algorithm to detect unexpected increases in frequency of reports, to enhance the ability to detect adverse events that manifest as increases in frequency, in particular quality defects, medication errors, and cases of abuse or misuse.

**Methods:**

An algorithm based on a negative binomial time‐series regression model run on 6 sequential observations prior to the monitored period was developed to forecast monthly counts of reports. A heuristic model to capture increases in counts when the previous 4 observations were null supplemented the regression. Count data were determined at drug‐event combination. Sensitivity analyses were run to determine the effect of different methods of pooling or stratifying count data. Positive retrospective detections and positive predictive values (PPVs) were determined.

**Results:**

The algorithm detected 8 of the 13 historical concerns, including all concerns of quality defects. The highest PPV (1.29%) resulted from increasing the lower count threshold from 3 to 5 and including literature reports in the counts. Both the regression model and the heuristic model components to the algorithm contributed to the detection of concerns. Sensitivity analysis indicates that stratification by commercial product reduces the PPV but suggests that pooling counts of related events may improve it.

**Conclusion:**

The results are encouraging and suggest that the algorithm could be useful for the detection of concerns that manifest as changes in frequency of reporting; however, further testing, including in prospective use, is warranted.

## 1 INTRODUCTION

Screening reports of suspected adverse drug reactions is a process through which data pertaining to different drug‐event combinations is provided and reviewed at regular intervals. This supports the decision on whether to perform a detailed review of the individual case safety reports, or simply reports, in view of confirming or dismissing the signal. A combination of data, including quantitative disproportionality methods, and qualitative elements^1^ is used to prioritise concerns based on risk‐proportionate ranking and performance.^2^ The criteria constitute a decision support tool.

Disproportionality methods do not provide a direct insight into temporal changes in frequency of reports. Although they can be applied over sequential periods,^3^ they are usually calculated from the cumulative data and hence are likely to be relatively insensitive to short‐term changes in reporting, which makes time‐trends harder to interpret.^4^ Thus, other tools are needed to help identify promptly changes in frequency.

Past attempts to define models to detect changes in frequency include an algorithm published by the Food and Drug Administration in 1992^5^ which required data on exposure, and thus information beyond that which is available in pharmacovigilance databases.

The Uppsala Monitoring Centre has also tested a modification of their Information Component algorithm applied to the identification of substandard medicines.^6^ The algorithm compares the Observed‐to‐Expected ratio of a country/year stratum to the Observed‐to‐Expected ratio of the other strata, using the Information Component. The authors selected a list of 78 terms they considered indicative of substandard medicines. This algorithm was not tested under the hypothesis that changes in the frequency of reports of harm can be a proxy of changes in quality.

The Sequential Probability Ratio Test, and variations, have also been proposed as a method to allow for multiple looks at accumulating data over time, more recently by Chan et al.^7^ The Sequential Probability Ratio Test is based on the difference, not the ratio, of observed‐to‐expected values. Expected values are obtained from a 2 × 2 contingency and include a hypothesised relative risk.

Other noteworthy algorithms include the work performed by DuMouchel et al who developed a statistical methodology to highlight excursions from baseline reporting using regression to model the time course of reports.^8^

From the earlier discussion, it becomes evident that at least 3 measures could be used to examine changes over time in pharmacovigilance databases: (1) changes in the disproportionality, (2) changes in proportion of reports of an event, and (3) changes in the count of reports of an event (time‐series regression).

The methods based on ratios have the advantage of not generating spurious signals when there are abrupt changes in the usage of the product or increased awareness affecting the overall reporting rate. However, they could generate attenuated signals when changes occur simultaneously in several drug‐event combinations. For instance, a quality defect leading to anaphylactic reaction may lead to increased reporting of angioedema, anaphylactic reaction, bradycardia, etc., and these could all fall below a signal threshold when considered separately.

The algorithm described in this paper was developed based on a regression modelling of counts of reports. This is simpler and more objective but may lead to a higher number of spurious signals from an increase in the use of a medicinal product or an artificial increase in the frequency of reporting. Whether this is an important disadvantage is determined by empirical evaluation of its performance in routine pharmacovigilance settings.

Certain types of events are likely to be reported in a reasonably concentrated period, such as product quality defects (QD), medication errors (ME), and abuse or misuse (A/M). Unintentional changes in the content of the medicinal product such as degradation of constituents and contamination result in deviation from the specified product quality. While not all quality issues will lead to a short‐term increase in reporting (for example a loss of potency of a vaccine could lead to reporting of lack of efficacy over a prolonged period), quality defects have shown this type of reporting pattern. A well‐studied example was the contamination of heparin with oversulfated chondroitin sulphate in 2008, which led to an increase in reports of allergic reactions.^9^ Initially, these reactions were not thought to be related to quality defects. Similarly, cases of abuse or misuse have led to increases in frequency of reports such as with ephedra,^10^ and medication errors have also manifested in the same way, such as with cabazitaxel.^11^

This analysis was aimed at validating a novel algorithm to detect unexpected increases in frequency (UIF) of reports to be used as an indicator of potential quality defects, medication errors, and abuse or misuse.

**KEY POINTS**

- A newly developed and tested algorithm to detect unexpected increases in frequency of reports of adverse events in EudraVigilance is presented.
- The algorithm correctly identified higher than expected frequencies of reports of several historical concerns related to quality defects, medication errors, and abuse and misuse.
- All quality defects were detected based solely on the reported harms, and not on terms related to product quality issues.

## 2 METHODS

### 2.1 Data source

The data were sourced from the European Union's central database of reports of suspected adverse drug reactions, EudraVigilance^12^ (EV). The reporting requirements of EV are detailed in the legislation and accompanying guideline on good pharmacovigilance practices Module VI.^13^

As with other algorithms used in routine signal detection, only data from the EV Post Marketing Module, excluding reports from studies, were used.

The count of reports was based on the receive date, the date when the report was received by the sender.

### 2.2 Algorithm

The algorithm consists of a negative binomial time‐series regression model developed in SAS® version 9.3. The Poisson distribution is widely used in pharmacovigilance to model count data; however, monthly counts of reports in pharmacovigilance databases tend to show over‐dispersed count data. This violates the assumption the variance equal to the mean; thus, a negative binomial distribution was preferred as it includes an extra parameter to model the over‐dispersion.

The unit of time was defined as a month. Let *t* indicate the unit of time, *t*_0_ indicate the monitored period, and T_6_ represent a period of 6 preceding months to the monitoring period (*t*_0_), such that T_6_ = {*t*_−6_, *t*_−5_,…,*t*_−1_} represents the 6 months prior to *t*_0_.

Let y be an observed count of reports per month, and y_0_ be the observed count of reports for the monitored period. The regression model is run based on the counts of the preceding 6 months to forecast the expected count at the monitoring period (ŷ) and respective confidence intervals. Let τ_n_ be a threshold of minimum count of reports. Previous research on report count thresholds for routine signal detection suggest that appropriate minimum counts are between 3 and 5 reports.^14^ Correspondingly, 2 thresholds were used to test the algorithm, 3 reports (τ_3_) and 5 reports (τ_5_).

**Figure 1:**
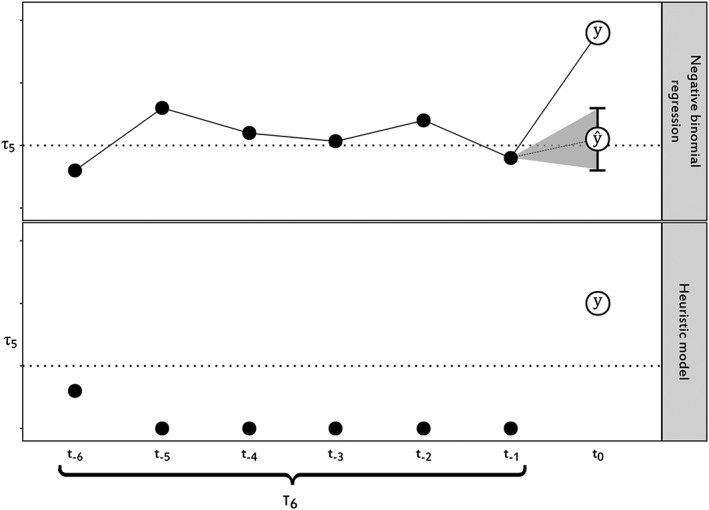
Hypothetical example of the elements of the algorithm. For the regression model, 6 sequential monthly counts are used to forecast the count ŷ at the monitoring period t_0_ and the confidence intervals. If the observed count y is higher than the upper bound of the ŷ estimate and the threshold has been achieved, an unexpected increase in frequency is detected. For the heuristic model, an unexpected increase in frequency is detected where y is higher than to the threshold τ

There is a possibility that sequential null counts occur; in such case, the regression model becomes less reliable. Hence, to allow for the detection of a sudden increase in counts following 4 sequential periods of null counts, a supplementary heuristic model was added to the algorithm. Where 4 or more of the immediately prior 6 observations were zero, the regression was not run, and the observed count y was compared with the threshold (τ). A theoretical example of the algorithm is presented in Figure 1.

A detection of an unexpected increase in frequency occurred where the observed count exceeded the upper bound of the forecast and the count exceed the threshold, or only when the observed count exceeded the threshold if the previous 4 observations were null.

### 2.3 Selection of historical controls

Historical concerns were defined as safety issues of the type that the algorithm is designed to detect. The European Pharmacovigilance Issues Tracking Tool (EPITT) was searched to collate all candidate events. Concerns that were detected by batch testing or before the administration of the product were excluded from the list of historical concerns as these were detected prior to human exposure, which led to a final list of 13 concerns (Table 1).

**Table 1:** List of historical concerns to test the algorithm to detect unexpected increases in frequency. All historical concerns of quality defects (QD), medication errors (ME), and abuse or misuse (A/M) were extracted from EPITT. The final list resulted from exclusion of concerns that were detected before human exposure. The events that were considered as indicative of the historical concern were all PTs grouped under the grouping terms (HLTs, HLGTs, and SMQs), eg, an increase in frequency of any PT of the SMQ embolic and thrombotic events would assist in detecting the embolic and thrombotic concern of 2010. The commercial product name refers to the products affected by the concern but other product names may exist

Substance	Commercial Product Name†	Index Date	Type	Concern	Events (All PTs Nested Under the Grouping Terms)
Human normal immunoglobulin	Octagam	23/08/2010	QD	Embolic and thrombotic events	Broad SMQ embolic and thrombotic events
Heparin	Heparin Rotexmedica	18/02/2008	QD	Hypotension, and serious allergic reactions, including some deaths	Broad SMQ anaphylactic reactions Broad SMQ hypersensitivity
Peritonial dialysis solutions	Baxter Extraneal, Baxter Nutrineal, Baxter Dianeal	16/10/2010	QD	Out‐of‐specification	HLT abdominal and gastrointestinal infections HLT peritoneal infections and febrile disorders
Epinephrine	Jext	07/11/2013	QD	Incorrect dose administered	Broad SMQ anaphylactic reactions Broad SMQ lack of efficacy/effect HLGT product use issues
Insulin aspart	Novomix	24/10/2013	QD	Out‐of‐specification (under and over dose)	HLGT glucose metabolism disorders (incl. diabetes mellitus) Broad SMQ Hyperglycaemia/new onset diabetes mellitus Broad SMQ Hypoglycaemia HLGT product use issues Broad SMQ lack of efficacy/effect
Fibrinogen‐containing solutions for sealant authorised for administration by spray application	Evecil, Quixil	21/06/2010	ME	Medication error leading to air embolism	Broad SMQ medication errors HLT non‐site specific embolism and thrombosis HLGT product use issues (for under and over dose)
Levetiracetam	Keppra	22/12/2015	ME	Incorrect dose administered	Broad SMQ medication errors HLGT neurological disorders NEC HLGT product use issues
Cabazitaxel	Jevtana	16/09/2013	ME	Reconstitution error	Broad SMQ medication errors HLGT product use issues
Adalimumab	Humira	13/09/2013	ME	Incorrect dose administered	Broad SMQ medication errors HLGT product use issues
Leuprorelin	Eligard	06/11/2013	ME	Incorrect dose administered	Broad SMQ medication errors HLGT product use issues
Loperamide	All	23/04/2015	A/M	Abuse leading to cardiac events, including QT prolongation	Broad SMQ medication errors HLGT product use issues (for under and over dose) HLT substance‐related disorders Broad SMQ drug abuse and dependence SMQ broad torsade de pointes/QT prolongation
Buprenorphine	All	21/03/2013	A/M	Abuse	HLT substance‐related disorders Broad SMQ drug abuse and dependence
Melatonin	All	17/10/2011	A/M	Abuse leading to euphoria, dependency, hallucinations, paranoia and schizophrenia.	Broad SMQ drug abuse and dependence Broad SMQ psychosis and psychotic disorders

The index date for the concerns was considered the date when the concern was first introduced in EPITT.

### 2.4 Events

The medical terminology used in pharmacovigilance regulatory activities and databases is the Medical Dictionary for Regulatory Activities (MedDRA).^15^ Preferred Terms (PTs) are the distinct descriptors in MedDRA and refer to a single medical concept for a symptom, sign, disease diagnosis, therapeutic indication, investigation, surgical or medical procedure, and medical social or family history characteristic. Related PTs are grouped into High Level Terms (HLTs) which are in turn subordinate to High Level Group Terms (HLGTs) and to System Organ Classes (SOCs).

In addition, MedDRA provides validated, standard sets of MedDRA PTs; these Standardised MedDRA Queries (SMQs) represent a variety of safety topics and are intended to aid in the identification and retrieval of potentially relevant individual case safety reports.

Each historical concern may have slightly different clinical manifestations, for instance, a contamination that causes thromboembolism may manifest as portal vein thrombosis or thrombophlebitis, etc. Hence, the concerns were defined at appropriate higher levels of MedDRA, and calculations were performed at PT level. This reflects the fact that an increase in any of the PTs grouped under a clinically suitable higher level of hierarchy or SMQ could help identify the historical concern.

Only PTs classified as important medical events (IME), ie, events that may not be immediately life‐threatening or result in death or hospitalisation but may jeopardise the patient or may require intervention to prevent one these outcomes,^16^ or as designated medical events^17^ were included.

MedDRA contains terms related to product quality issues that were not included in the case definition of the historical concerns related to quality defects as these would have been highlighted and would have triggered appropriate regulatory action prior or concomitantly to reporting to EV. The underlying premise of the algorithm is that the detection of a quality defect can be achieved through detection of changes in the frequency of harms. In the case of medication errors or abuse or misuse, regulatory action is not programmatically set; hence, terms related to these were included.

### 2.5 Positive identification

An increase in frequency was considered a true positive if it occurred 1 year prior or 6 months after the index date of a MedDRA PT related to the reported concern. An increase in frequency was considered a false positive if it occurred outside the period or occurred to MedDRA terms not related to the concern.

The window of time was defined empirically to take into account the fact that these historical examples were collected in the absence of a tool to monitor real‐time increases in frequency. It is therefore possible that they only became evident over a relatively long period. This would include the actual detection of the issue and additional regulatory timelines, such that the entry date in EPITT may have been several months after the event. As the event may persist beyond the initial detection, due to a wash‐out of products still in the market, the time window was extended past the entry date in EPITT.

### 2.6 Methodological choices for determining monthly count data

The main analysis focused on testing the algorithm in conditions that simulate the routine monitoring of drug safety concerns: monthly counts were determined as the count of an event, at PT level, for a substance, in a calendar month.

Spontaneous reports include reports that stem from published scientific literature and including them in the monthly report count may hypothetically have an effect on the performance of the algorithm as they are associated with higher rates of duplicate reporting.^18^ To assess this effect, the analysis was run including and excluding reports from the literature.

Furthermore, it is known that different approaches to pooling or stratifying counts of reports may influence the results of the algorithm. This concern can derive from the level of MedDRA chosen^19^ but also from the granularity at which the drug is identified, namely if it is stratified by commercial product name. Sensitivity analyses were run to assess the performance of changing these parameters.

### 2.7 Metrics

In the absence of a gold standard that would allow a comparative analysis, the positive predictive value (PPV) was defined as the ratio between all UIF for each historical concern and all UIF in the dataset for the substances included in the list of historical concerns.

## 3 RESULTS

The algorithm developed correctly detected 8 of the 13 historical concerns, including all quality defects (Table 2).

**Table 2:** Performance of the algorithm on counts at MedDRA PT and substance level. The table shows the performance of the algorithm when calculating counts of reports at MedDRA PT and substance level, simulating routine signal detection procedures. The effect of excluding literature reports and using different thresholds is presented

	Counts at MedDRA PT and Substance			
	Including reports from literature		Excluding reports from Literature	
	Threshold τ_3_	Threshold τ_5_	Threshold τ_3_	Threshold τ_5_
Average PPV model	1.03%	1.29%	0.82%	1.02%
*Time‐series regression model*	*1.36%*	*1.31%*	*0.96%*	*1.00%*
*Heuristic model*	*0.88%*	*1.24%*	*0.76%*	*1.01%*
Detections	8/13	8/13	8/13	7/13
Detections by type	QD 5/5	QD 5/5	QD 5/5	QD 4/5
	ME 1/5	ME 1/5	ME 1/5	ME 1/5
	A/M 2/3	A/M 2/3	A/M 2/3	A/M 2/3

Abbreviations: A/M, abuse or misuse; ME, medication error; QD, quality defect.

The PPVs ranged between 1.03% and 1.29% in the analysis that included reports from literature in the monthly counts and between 0.82% and 1.03% in the analysis that excluded these reports. Increasing the lowest count threshold from 3 to 5 improved the PPVs in both analyses, at no expense in the number of concerns detected.

**Figure 2:**
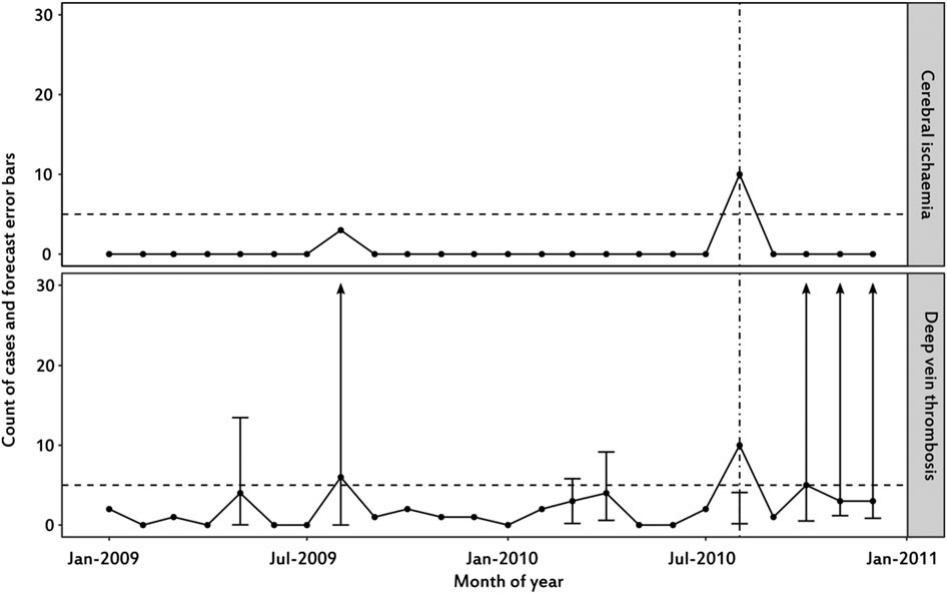
Graphical representation of the identification of historical concerns with counts of cases at MedDRA PT and substance level, including literature, for 2 reactions (cerebral infraction and deep vein thrombosis) reported to human normal immunoglobulin. Period shown between January 2009 and December 2010. The dashed horizontal line depicts the minimum count threshold (5 in the example). The dashed‐dotted vertical line indicates an unexpected increase in frequency. The plot for deep vein thrombosis illustrates how forecasts are only produced when the observed counts exceed the minimum threshold with an unexpected increase in frequency detected in August 2010. The plot concerning cerebral ischaemia illustrates how in the presence of several null observations detection of unexpected increases in frequency relies on the heuristic model

The time‐series regression element of the algorithm had higher PPVs than the heuristic model element, but both contributed to the detection of the concerns. A graphical example of the application of the algorithm in reports of human normal immunoglobulin to 2 different PTs, with manifestly different counts (Figure 2), illustrates the application of different components of the algorithm.

The sensitivity analysis (supplementary material) showed that stratification by commercial product name and a reduction in the number of monthly observations from 6 to 3 reduce the PPVs, whereas pooling counts of reports based on the events was likely to improve the PPVs.

## 4 DISCUSSION

The analyses showed that 8 out of 13 historical concerns were detected using the algorithm. These results are promising, particularly considering that all quality defects were detected solely through the increase in PTs related to harm rather than through PTs related to product quality defects. This is important, as these are the concerns that are more likely to go unnoticed—quality defects that are only reported as harm—particularly if the reported PTs refer to safety concerns already known for the medicinal product, such as those stemming from overdose.

The algorithm also detected the majority of concerns of abuse or misuse. This supports the potential use of the algorithm to detect the acute events of abuse or misuse that concentrate in short periods; however, the dynamics of reporting of cases of abuse or misuse needs further research.

It is unclear why the algorithm fared worse in detecting medication errors. It may be due to the fact that these events have only recently been formally included under the mandate of regulatory pharmacovigilance^20^ or that an important amount of data is collected elsewhere, such as by poison control centres.

These concerns may have serious public health consequences, but fortunately, they are fairly rare, and thus only a few are available to use as historical concerns, which affects generalizability. To sidestep this problem, the study could have used dummy data; however, it would have produced an artificial setting which would not allow an understanding of the aptitude of the algorithm to detect true concerns in real world data.

Whereas, it would have been preferable to have used a gold standard or established algorithm to compare the results to, research in this area is limited and typically run by pharmaceutical companies and regulatory authorities looking to expand their drug safety monitoring toolkit, which may explain the fact that there are relatively few publications on the topic.

The algorithm tested differed from previous research. It was deliberately restricted to a simple time‐series regression complemented with a heuristic model, it used a short time window of 6 months, to allow quick roll‐out for new medicinal products, it does not require data from other products or determining hypothesised relative risks, and it interrogates data in a manner fundamentally different to current algorithms.

The sensitivity analysis provides useful insight that pooling counts at higher levels of MedDRA may improve the PPV, but the dynamics of this effect are unclear as at higher levels contradictory terms may be combined, such as for the HLGT Product use issues that includes “overdose” and “underdose”, which would be distinct quality defects. Future research is needed to understand if other levels might achieve better results, including using SMQs or bespoke groupings of terms.

The resulting PPVs might be construed as low; however, it should be noted that algorithms in pharmacovigilance act as decision support tools and expert review is always performed, and hence a relatively larger number of false positives can be considered acceptable as a trade‐off to enhancing the toolkit of safety monitoring. At any rate, additional research is routinely performed to both adapt the pharmacovigilance toolkit to regulatory changes and to improve its performance and efficiency.

The highest PPV (1.29%) was achieved by using a minimum count threshold of 5 reports and including reports from the literature in the counts. A balance is needed in setting the minimum count threshold. Increasing it is likely to reduce the number of false positives, and thereof increase the PPV: this is seen in the results of the analyses. However, the threshold should not be as large as to require an unduly number of events to occur prior to detection, especially considering that under‐reporting means an unknown fraction of these events is never reported.

Whereas it is possible that including literature reports will lead to false positives due to a spurious increase in the frequency, the exclusion of these from the counts seems to have an important depletory effect on the PPV.

The regression model did not include spatial‐temporal adjustment. Theoretically, it cannot be assumed that the geographical distribution of any of these events is different than for any other reaction. A contamination in the water for sterile injections in a European‐wide production facility, for instance, would lead to events across countries, whereas the abuse of a psychoactive product is equally unlikely to be bound to a location, unless different access restrictions exist. At any rate, by pooling the counts of reports from different countries it is still be possible to understand a posteriori if the concern is geographically contained. On the other hand, to adjust for seasonality, years of observations would be needed, the downside is that it would postpone the implementation the algorithm to years after the introduction of a new product to the market.

## 5 CONCLUSION

The algorithm developed to detect UIF allowed the detection of most historical concerns. Whereas the algorithm warrants further testing, including in prospective use, the results suggest it could be useful for the detection of concerns that manifest as changes in frequency of reporting.

## ETHICS STATEMENT

The authors state that no ethical approval was needed.

## CONFLICT OF INTEREST

The authors declare no conflict of interest.

## DISCLAIMER

The views expressed in this article are the personal views of the authors and may not be understood or quoted as being made on behalf of or reflecting the position of the European Medicines Agency or one of its committees, working parties, or national competent authorities.

## FUNDING

This initiative received no specific grant from any funding agency in the public, commercial, or not‐for‐profit sectors.

## DATA SHARING

EudraVigilance data are available subject to the “EudraVigilance access policy for medicines for human use” available at http://www.ema.europa.eu/ema/index.jsp?curl=pages/regulation/general/general_content_000674.jsp.

MedDRA® the Medical Dictionary for Regulatory Activities terminology is the international medical terminology developed under the auspices of the International Conference on Harmonisation of Technical Requirements for Registration of Pharmaceuticals for Human Use (ICH). MedDRA® trademark is owned by IFPMA on behalf of ICH.

## Supporting information

Table S1: Performance of the algorithm pooling counts at MedDRA PT by Commercial product name. The table shows the performance of the algorithm when calculating counts of reports at MedDRA PT and Commercial product name. The effect of excluding literature reports and using different thresholds is presented. Two quality defects historical concerns could not be calculated at commercial product name as insufficient reports specified product name. Cases of abuse or misuse are not product specific and were not considered in the analyses that stratified by commercial product name.
Table S2: Performance of the algorithm pooling counts of all reports (all PTs) by Substance. The table shows the performance of the algorithm when calculating counts of reports by pooling all adverse reactions (ie, all MedDRA PTs) by Substance. The effect of excluding literature reports and using different thresholds is presented.
Table S3: Performance of the algorithm pooling counts of all reports (all PTs) by Commercial product name. The table shows the performance of the algorithm when calculating counts of reports by pooling all adverse reactions (ie, all MedDRA PTs) by Commercial product name. The effect of excluding literature reports and using different thresholds is presented. Two quality defects historical concerns could not be calculated at commercial product name as insufficient reports specified product name. Cases of abuse or misuse are not product specific and were not considered in the analyses that stratified by commercial product name.
Table S4: Performance of the algorithm on counts at MedDRA PT and substance level, using 3 months observations rather than 6 months of observations. The table shows the performance of the algorithm when calculating counts of reports at MedDRA PT and Substance level using 3 months of observations, mimicking routine signal detection procedures. The effect of excluding literature reports and using different thresholds is presented.
