# Supplementary material for: An algorithm to detect unexpected increases in frequency of reports of adverse events in EudraVigilance
Source: Pharmacoepidemiol Drug Saf. 2017 Nov 16;27(1):38–45. doi: 10.1002/pds.4344 (PMC5765515; doi:10.1002/pds.4344)
Supplement: Supplementary file 1 — Table S1: Performance of the algorithm pooling counts at MedDRA PT by Commercial product name. The table shows the performance of the algorithm when calculating counts of reports at MedDRA PT and Commercial product name. The effect of excluding literature reports and using different thresholds is presented. Two quality defects historical concerns could not be calculated at commercial product name as insufficient reports specified product name. Cases of abuse or misuse are not product specific and were not considered in the analyses that stratified by commercial product name. Table S2: Performance of the algorithm pooling counts of all reports (all PTs) by Substance. The table shows the performance of the algorithm when calculating counts of reports by pooling all adverse reactions (ie, all MedDRA PTs) by Substance. The effect of excluding literature reports and using different thresholds is presented. Table S3: Performance of the algorithm pooling counts of all reports (all PTs) by Commercial product name. The table shows the performance of the algorithm when calculating counts of reports by pooling all adverse reactions (ie, all MedDRA PTs) by Commercial product name. The effect of excluding literature reports and using different thresholds is presented. Two quality defects historical concerns could not be calculated at commercial product name as insufficient reports specified product name. Cases of abuse or misuse are not product specific and were not considered in the analyses that stratified by commercial product name. Table S4: Performance of the algorithm on counts at MedDRA PT and substance level, using 3 months observations rather than 6 months of observations. The table shows the performance of the algorithm when calculating counts of reports at MedDRA PT and Substance level using 3 months of observations, mimicking routine signal detection procedures. The effect of excluding literature reports and using different thresholds is presented. [file PDS-27-38-s001.docx]

**Supplementary material**

**Table 1: Performance of the algorithm pooling counts at MedDRA PT by Commercial product name.** The table shows the performance of the algorithm when calculating counts of reports at MedDRA PT and Commercial product name. The effect of excluding literature reports and using different thresholds is presented. Two quality defects historical concerns could not be calculated at commercial product name as insufficient reports specified product name. Cases of abuse or misuse are not product specific and were not considered in the analyses that stratified by commercial product name.

|  | Counts at MedDRA PT and Commercial product name | | | |
| --- | --- | --- | --- | --- |
|  | Including reports from literature | | Excluding reports from literature | |
|  | **Threshold τ_3_** | **Threshold τ_5_** | **Threshold τ_3_** | **Threshold τ_5_** |
| **Average PPV model** | 0.63% | 0.65% | 0.59% | 0.62% |
| ***Time-series regression model*** | *0.53%* | *0.29%* | *0.56%* | *0.31%* |
| ***Heuristic model*** | *0.67%* | *1.06%* | *0.60%* | *1.00%* |
| **Detections** | 4/8^†^ | 2/8^†^ | 3/8^†^ | 2/8^†^ |
| **Detections by type** | QD 2/3  ME 2/5  A/M - | QD 2/3  ME 0/5  A/M - | QD 2/3  ME 1/5  A/M - | QD 2/3  ME 0/5  A/M - |
| QD – Quality defect  ME – Medication Error  A/M – Abuse or Misuse | | | | |

**Table 2: Performance of the algorithm pooling counts of all reports (all PTs) by Substance.** The table shows the performance of the algorithm when calculating counts of reports by pooling all adverse reactions (i.e. all MedDRA PTs) by Substance. The effect of excluding literature reports and using different thresholds is presented.

|  | Counts of all reports (all MedDRA PTs pooled) by Substance | | | |
| --- | --- | --- | --- | --- |
|  | Including reports from literature | | Excluding reports from literature | |
|  | **Threshold τ_3_** | **Threshold τ_5_** | **Threshold τ_3_** | **Threshold τ_5_** |
| **Average PPV model** | 16.84 % | 16.75% | 15.26% | 15.59% |
| ***Time-series regression model*** | *17.10%* | *16.84%* | *15.14%* | *15.38%* |
| ***Heuristic model*** | *0.00%* | *0.00%* | *0.20%* | *0.25%* |
| **Detections** | 13/13 | 13/13 | 13/13 | 12/13 |
| **Detections by type** | QD 5/5  ME 5/5  A/M 3/3 | QD 5/5  ME 5/5  A/M 3/3 | QD 5/5  ME 5/5  A/M 3/3 | Q/D 5/5  ME 5/5  A/M 2/3 |
| QD – Quality defect  ME – Medication Error  A/M – Abuse or Misuse | | | | |

**Table 3: Performance of the algorithm pooling counts of all reports (all PTs) by Commercial product name**. The table shows the performance of the algorithm when calculating counts of reports by pooling all adverse reactions (i.e. all MedDRA PTs) by Commercial product name. The effect of excluding literature reports and using different thresholds is presented. Two quality defects historical concerns could not be calculated at commercial product name as insufficient reports specified product name. Cases of abuse or misuse are not product specific and were not considered in the analyses that stratified by commercial product name.

|  | Counts of all reports (all MedDRA PTs pooled) by Commercial product name | | | |
| --- | --- | --- | --- | --- |
|  | Including reports from literature | | Excluding reports from literature | |
|  | **Threshold τ_3_** | **Threshold τ_5_** | **Threshold τ_3_** | **Threshold τ_5_** |
| **Average PPV model** | 18.75% | 16.88% | 18.18% | 17.81% |
| ***Time-series regression model*** | *18.42%* | *17.57%* | *18.92%* | *18.31%* |
| ***Heuristic model*** | *0.25%* | *0.00%* | *0.00%* | *0.00%* |
| **Detections** | 7/8^†^ | 6/8 | 6/8^†^ | 6/8 |
| **Detections by type** | QD 2/3  ME 5/5  A/M - | QD 2/3  ME 4/5  A/M - | QD 2/3  ME 4/5  A/M - | QD 2/3  ME 4/5  A/M - |
| QD – Quality defect  ME – Medication Error  A/M – Abuse or Misuse | | | | |

**Table 4: Performance of the algorithm on counts at MedDRA PT and substance level, using three months observations rather than six months of observations.** The table shows the performance of the algorithm when calculating counts of reports at MedDRA PT and Substance level using three months of observations, mimicking routine signal detection procedures. The effect of excluding literature reports and using different thresholds is presented.

|  | Counts at MedDRA PT and Substance | | | |
| --- | --- | --- | --- | --- |
|  | Including reports from literature | | Excluding reports from literature | |
|  | **Threshold τ_3_** | **Threshold τ_5_** | **Threshold τ_3_** | **Threshold τ_5_** |
| **Average PPV model** | 0.94% | 1.23% | 0.85% | 1.17% |
| ***Time-series regression model*** | *1.09%* | *1.22%* | *1.05%* | *1.38%* |
| ***Heuristic model*** | *0.88%* | *1.23%* | *0.77%* | *1.00%* |
| **Detections** | 8/13 | 8/13 | 8/13 | 7/13 |
| **Detections by type** | QD 5/5  ME 1/5  A/M 2/3 | QD 5/5  ME 1/5  A/M 2/3 | QD 5/5  ME 1/5  A/M 2/3 | QD 4/5  ME 1/5  A/M 2/3 |
| QD – Quality defect  ME – Medication Error  A/M – Abuse or Misuse | | | | |
